# Study on Rapid Non-Destructive Detection Method of Corn Freshness Based on Hyperspectral Imaging Technology

**DOI:** 10.3390/molecules29132968

**Published:** 2024-06-21

**Authors:** Yurong Zhang, Shuxian Liu, Xianqing Zhou, Junhu Cheng

**Affiliations:** 1 School of Food and Strategic Reserves, Henan University of Technology, Zhengzhou 450001, China; yurongzh@163.com (Y.Z.); lshuxian123@163.com (S.L.); 2 Engineering Research Center of Grain Storage and Security of Ministry of Education, Zhengzhou 450001, China; 3 Henan Provincial Engineering Technology Research Center on Grain Post Harvest, Zhengzhou 450001, China; 4 School of Food Science and Engineering, South China University of Technology, Guangzhou 510641, China

**Keywords:** corn, fatty acid values, freshness, hyperspectral imaging, chemical information visualization

## Abstract

(1) Background: To achieve the rapid, non-destructive detection of corn freshness and staleness for better use in the storage, processing and utilization of corn. (2) Methods: In this study, three varieties of corn were subjected to accelerated aging treatment to study the trend in fatty acid values of corn. The study focused on the use of hyperspectral imaging technology to collect information from corn samples with different aging levels. Spectral data were preprocessed by a convolutional smoothing derivative method (SG, SG1, SG2), derivative method (D1, D2), multiple scattering correction (MSC), and standard normal transform (SNV); the characteristic wavelengths were extracted by the competitive adaptive reweighting method (CARS) and successive projection algorithm (SPA); a neural network (BP) and random forest (RF) were utilized to establish a prediction model for the quantification of fatty acid values of corn. And, the distribution of fatty acid values was visualized based on fatty acid values under the corresponding optimal prediction model. (3) Results: With the prolongation of the aging time, all three varieties of corn showed an overall increasing trend. The fatty acid value of corn can be used as the most important index for characterizing the degree of aging of corn. SG2-SPA-RF was the quantitative prediction model for optimal fatty acid values of corn. The model extracted 31 wavelengths, only 12.11% of the total number of wavelengths, where the coefficient of determination R_P_^2^ of the test set was 0.9655 and the root mean square error (RMSE) was 3.6255. (4) Conclusions: This study can provide a reliable and effective new method for the rapid non-destructive testing of corn freshness.

## 1. Introduction

Corn is one of the most important food crops in the world, accounting for about one-third of China’s total grain production. But, as a seasonal crop, storage is an essential step before corn can be eaten or processed [1]. During storage, corn is susceptible to mold, mites, pests, and other environmental factors that can lead to a loss of quality [2]. Therefore, it is important to quickly and accurately identify the storage quality of corn. It has been demonstrated that fatty acid content and composition have a significant impact on corn storage quality and eating quality, and the fatty acid value of corn increases with the storage time [3,4]. Changes in fatty acid values are influenced by a variety of factors, such as temperature, humidity, storage time, oxygen concentration, and light. Among them, temperature and humidity are the main factors affecting the variation in fatty acid values [5,6]. On the one hand, under conditions of high temperature and humidity, the water within the corn evaporates, causing the oxidation of oils and fats and consequently increasing the fatty acid value; on the other hand, aldehydes and ketones will be produced under this condition, which are harmful to the human body. In China, in the national standard of GB/T 20570-2015 (corn storage quality judgment rules) [7], it is stipulated that when the fatty acid value is ≤65 mg KOH/100 g, it is suitable for storage; when the fatty acid value is ≤78 mg KOH/100 g, it is not suitable for storage; when the fatty acid value is >78 mg KOH/100 g, it is seriously not suitable for storage. Therefore, the fatty acid value can indirectly reflect the vitality of corn kernels and is also used as one of the important indexes for determining the newness of corn.

In recent years, hyperspectral imaging technology has been applied for raw grains such as wheat, corn, rice, and soybeans to achieve non-destructive and rapid detection [8,9,10,11]. Hyperspectral imaging technology synthesizes the advantages of spectral technology and imaging technology, which is a more advanced non-destructive testing technology [12]. For example, the accuracy of the k-nearest neighbor (KNN) model was 93.71% for barley variety identification using the hyperspectral imaging technique [13]. Hyperspectral imaging was used to rapidly analyze the moisture content of corn kernels with a predicted correlation coefficient (r) of 0.9064 [14]. An unsupervised redundant co-clustering algorithm (FCM-SC) based on multicenter fuzzy c-mean (FCM) clustering and spectral clustering (SC) was proposed by Kang et al. This algorithm was capable of describing the complex structure of corn kernel mold distribution and exhibited higher stability, anti-interference, generalization, and accuracy than supervised classification models [15].

However, using hyperspectral imaging technology combined with algorithms to predict corn freshness has rarely been reported. Therefore, this study focused on the changes in corn fatty acid value during accelerated aging and the quantitative prediction of corn fatty acid value based on hyperspectral imaging technology to establish the best prediction model. This model can predict the grade of corn freshness of aging and the visualization of corn fatty acid value by the best model. This study can realize the rapid non-destructive testing of corn freshness and provide a more rapid, convenient, and non-destructive method for the detection and monitoring of grain reserves.

## 2. Results and Discussion

### 2.1. Changes in Fatty Acid Values of Corn during Aging and Sample Set Partitioning

**Figure 1:**
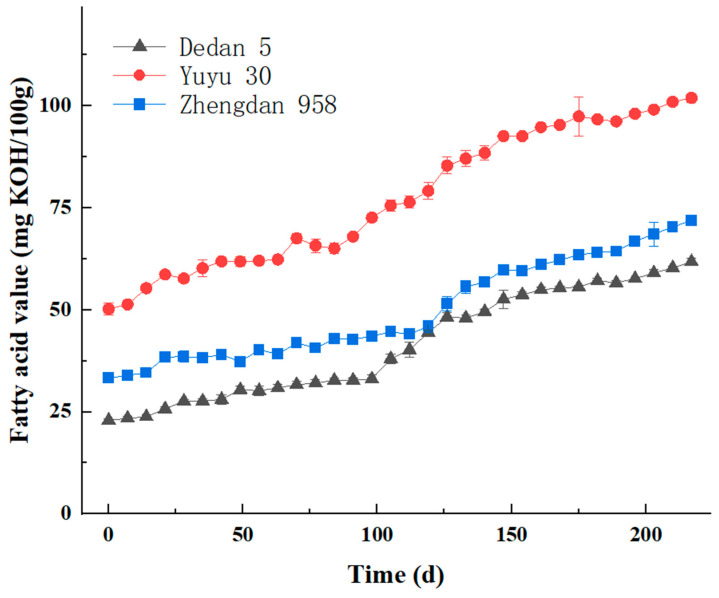
Changes in fatty acid values during corn aging.

The overall pattern of changes in fatty acid values during the aging of corn is shown in Figure 1. Due to a large number of samples, each point in the figure represents the average of the samples that were taken.

As shown in Figure 1, with the prolongation of the aging time, all three varieties of corn showed an overall increasing trend, which is consistent with the previous reports studying the changes in fatty acid values of corn during storage [16,17,18]. Temba, M. C. et al. found that the rate of change in FFA significantly (*p* ≤ 0.05) increased with increasing storage time. The fatty acid value of Dedan 5 increased slowly, from 22.96 mg KOH/100 g to 61.78 mg KOH/100 g, an increase of 38.82 mg KOH/100 g. The fatty acid value of Yuyu 30 increased the most drastically, from 50.22 mg KOH/100 g to 101.92 mg KOH/100 g, an increase of 51.7 mg KOH/100 g. The fatty acid value of Zhengdan 958 increased to the same extent as that of Dedan 5, from 33.25 mg KOH/100 g to 71.91 mg KOH/100 g, an increase of 38.66 mg KOH/100 g. Different varieties of corn resulted in some differences in the initial values of fatty acid values and the drastic degree of increase. Only a few minor fluctuations up and down occurred during the aging process. This may be due to the short sampling interval, the temperature and humidity errors caused by the different storage locations of the corn samples, the water content of the corn samples, the different metabolic patterns of the corn samples at each stage of the aging process, and the differences in physiological activity of the corn kernels at the post-ripening stage [19,20]. However, as a whole, there is a very good correlation between the fatty acid value and aging time of corn. Therefore, the fatty acid value of corn can be used as the most important index for characterizing the degree of aging of corn.

Based on the sample set partitioning based on the joint x-y distance (SPXY) algorithm, the sample set of 363 samples was divided according to a ratio of the training set to test set of 2:1, and 242 samples of the training set and 121 samples of the test set were obtained. In this way, the training set measurement value range (22.68 mg KOH/100 g~102.93 mg KOH/100 g) of fatty acid values can be included in the test set measurement value range (23.48 mg KOH/100 g~98.49 mg KOH/100 g), and the maximum, minimum, mean, and standard deviation values can be calculated [1]. The calculated results are shown in Table 1.

### 2.2. Data Preprocessing and Extraction of Characteristic Bands

**Figure 2:**
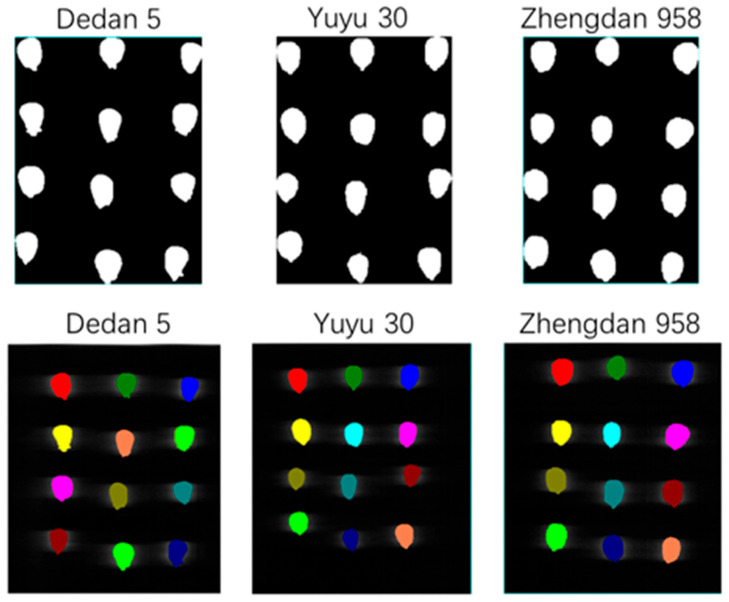
Map of corn seed mask and region of interest in selected results.

The ENVI (5.3) software was used to select the mask and region of interest for the acquired hyperspectral image information, as shown in Figure 2. And, the corresponding spectral information was extracted, and the extracted spectral information was averaged and recorded and saved.

**Figure 3:**
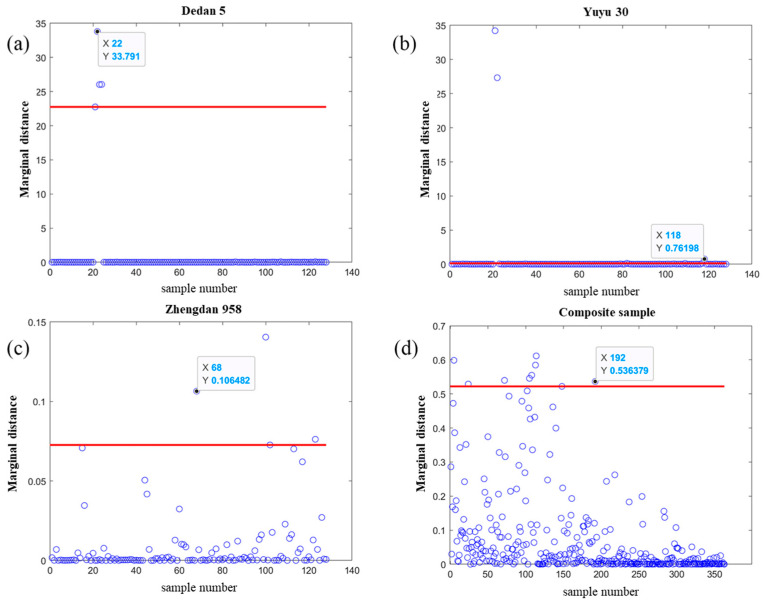
Anomalous spectra rejected by marginal distance. (**a**) Outliers were removed from Dezhou 5; (**b**) outliers were removed from Yuyu 30; (**c**) outliers were removed from Zhengzhou 958; (**d**) outliers were removed from mixed samples.

The MATLAB (2020b) software was used to classify, cluster, and detect anomalies in the sample information using the Mahalanobis distance method [21,22]. In classification and clustering algorithms (such as K-means and nearest neighbor classifiers), using the Mahalanobis distance can improve the accuracy of classification and clustering, especially when feature distributions are uneven or correlated. The distribution of the marginal distance is shown in Figure 3.

In Figure 3, the red color is the threshold value, those greater than or equal to the threshold value are abnormal values, and those less than the threshold value are normal values. Figure 3a shows the total number of 128 samples for Dedan 5, excluding 4 abnormal samples, and the total number of rejected samples is 21, 22, 23, and 24. Figure 3b shows the estimated number of 128 samples for Yuyu 30, excluding 4 abnormal samples, and the total number of rejected samples is 21, 22, 82, and 118. Figure 3c shows 128 estimated samples of Zhengdan 958, with 4 abnormal samples removed, and a total of 68, 100, 102, and 123 samples removed. Figure 3d shows the estimation of 372 mixed samples of three different varieties of corn, excluding abnormal samples, totaling 9 abnormal samples that total 5, 24, 72, 105, 108, 113, 114, and 192. The remaining samples after elimination totaled 363.

**Figure 4:**
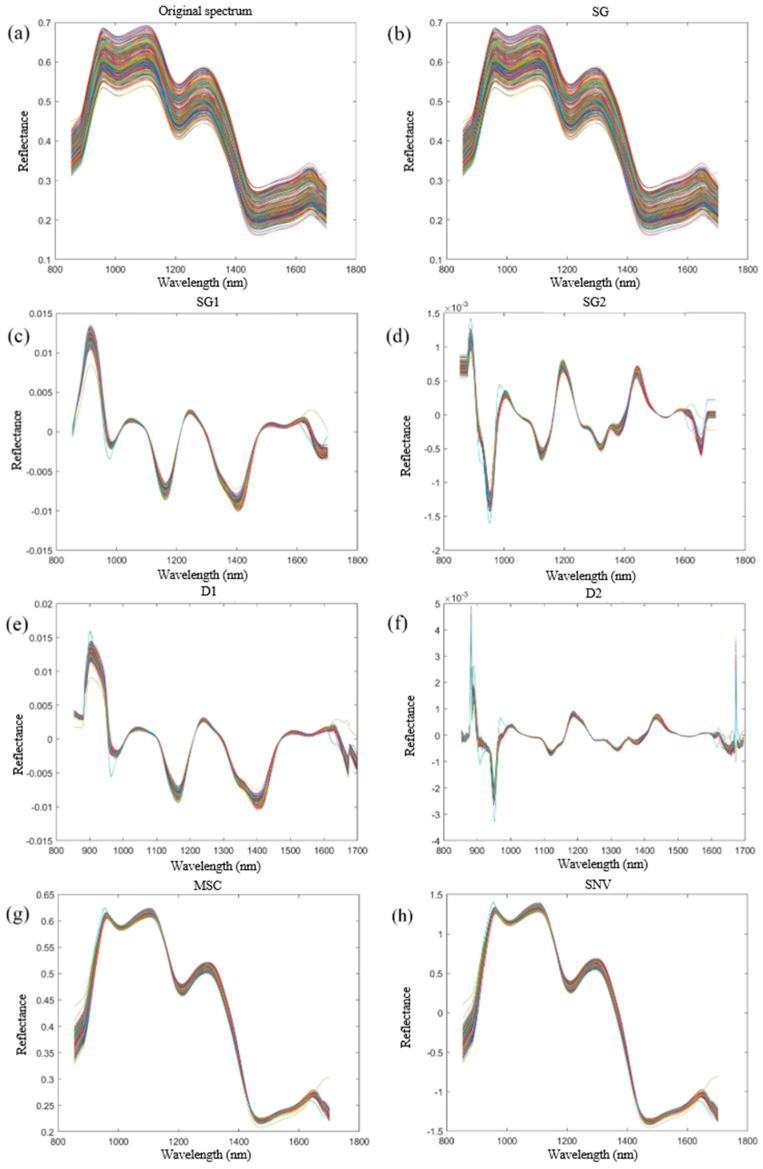
Raw and seven preprocessed spectral images. (**a**) The original spectral image; (**b**) SG preprocessed spectral images; (**c**) SG1 preprocessed spectral images; (**d**) SG2 preprocessed spectral images; (**e**) D1 preprocessed spectral images; (**f**) D2 preprocessed spectral images; (**g**) MSC preprocessed spectral images; (**h**) SNV pretreatment of spectral images.

The average spectral data of the remaining samples (363 in total) were extracted, and the data were processed and analyzed using seven preprocessing methods. The results are shown in Figure 4. Figure 4a shows the raw spectral image. During storage, corn underwent processes such as lipid oxidation and protein denaturation, alongside changes in its physical structure and moisture content. These factors collectively contributed to varying degrees of alteration in the intensity, shape, and displacement of spectral absorption peaks. And, it can be seen that there were many noises and fluctuations. Many scholars preprocessed spectral data before analyzing spectral data so as to significantly improve the quality and analysis effect of spectral data, making spectral analysis more accurate, efficient, and reliable. Different pretreatments had different effects. Therefore, the spectrum needed to be preprocessed in order to improve the signal-to-noise ratio and the reliability of the data [23,24]. Figure 4b shows the spectral image after smoothing (SG) processing, which removed significant invalid information such as systematic noise and retained the detailed information of the spectrum, resulting in better spectral curve smoothing and continuity. This method is suitable for the case where the spectral peaks are relatively flat [25]. The graph in Figure 4c shows the spectral image after smoothed first-order derivative (SG1) processing, which highlights the slope information of the peaks and indicates the speed of the band change and location of the extremes [26]. The Figure 4d graph shows the spectral image after smoothed second-order derivation (SG2) processing, which provides the concave and convex information of the spectral curve and helps to separate the secondary absorption peaks and valleys generated by the overlapping absorption region. This method highlights the curvature information of the peaks and also removes the flat-topping phenomenon that may occur after smoothing [27]. Figure 4e shows the spectral image after first-order derivation (D1) processing. This method highlights the rate of change in the peaks and facilitates peak detection and identification [28]. Figure 4f shows the spectral image after the second-order derivative (D2) processing, which highlights the curvature of the peaks and facilitates peak detection and identification [29]. Figure 4g shows the spectral image after MSC preprocessing, which removes the effect of scattering on the spectrum caused by impurities and clutter in the sample and improves the accuracy of the prediction model [30]. Figure 4h shows the spectral image after SNV preprocessing, which removes the effect of scaling in the spectra, reduces the error caused by scattering between the calibration samples, and improves the comparability and reliability of the data [31,32].

The total number of optimal bands extracted from the CARS characteristic wavelengths in descending order was RAW-CARS > D2-CARS > SG-CARS > D1-CARS > SNV-CARS > SG2-CARS > SG1-CARS > MSC-CARS. The optimal bands were 62, 56, 51, 48, 42, 40, 38, and 31, accounting for 24.22%, 21.88%, 19.92%, 18.75%, 16.41%, 15.63%, 14.84%, and 12.11% of the total number of wavelengths, respectively.

The total number of optimal wavelengths extracted from the SPA characteristic wavelengths in descending order was SG2-SPA > D2-SPA > MSC-SPA > SNV-SPA > SG1-SPA = D1-SPA > RAW-SPA = SG-SPA, and the optimal wavelengths were 31, 25, 24, 23, 20, 20, 10, and 10, accounting for 12.11%, 9.77%, 9.38%, 8.98%, 7.81%, 7.91%, 3.91%, 3.91%, and 3.91% of the total number of wavelengths, respectively.

### 2.3. Model Construction for Predicting Fatty Acid Values of Corn Based on Neural Network

Full-band and eigen-band modeling was based on neural network (BP) fatty acid values combined with RAW, SG, SG1, SG2, D1, D2, MSC, and SNV preprocessed spectra. The coefficient of determination (R^2^), root mean square error (RMSE), and mean absolute percentage error (MAPE) of the models in the training and test sets are shown in Table 2.

As can be seen from Table 2, the highest training set coefficient of determination (R^2^) was the SG1-SPA-BP model, and the highest test set coefficient of determination was the MSC-SPA-BP model. Because the test set coefficient of determination can be better used to determine the best model [33], the best predictive model was the MSC-SPA-BP model.

**Figure 5:**
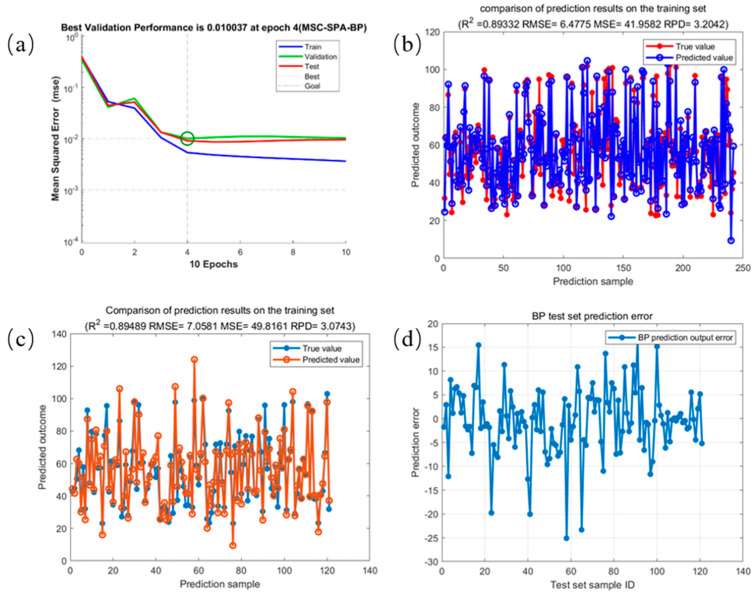
Results of the analysis of fatty acid values of corn based on the best MSC−SPA−BP model. (**a**) MSC−SPA−BP training process; (**b**) MSC−SPA−BP test set prediction error; (**c**) comparison of prediction results of MSC−SPA−BP training set; (**d**) comparison of prediction results of MSC−SPA−BP test set.

The results of the fatty acid value analysis of corn based on the best feature band MSC-SPA-BP model are shown in Figure 5. Figure 5a gives the BPNN training process using the MSC preprocessed optimized SPA feature band spectra, and the blue, green, and red curves represent the mean square error iteration curves of the training set, validation set, and test set, respectively. It can be seen that, after processing with MSC-SPA, the network had a preset number of 100 iterations, and the best model performance was achieved when the number of iterations was 4. Figure 5b shows the training set modeling output of the BP neural grid for fatty acid values with a training set coefficient of determination (R_C_^2^) of 0.8933 and RMSE_C_ of 6.4475 for the prediction of fatty acid values of corn samples using a trained BPNN. Figure 5c shows the output of the BPNN for the test set prediction of corn samples with a test set coefficient of determination (R_P_^2^) of 0.8949 and RMSE_P_ of 7.0581. Figure 5d shows the result of the prediction error for the test set.

**Figure 6:**
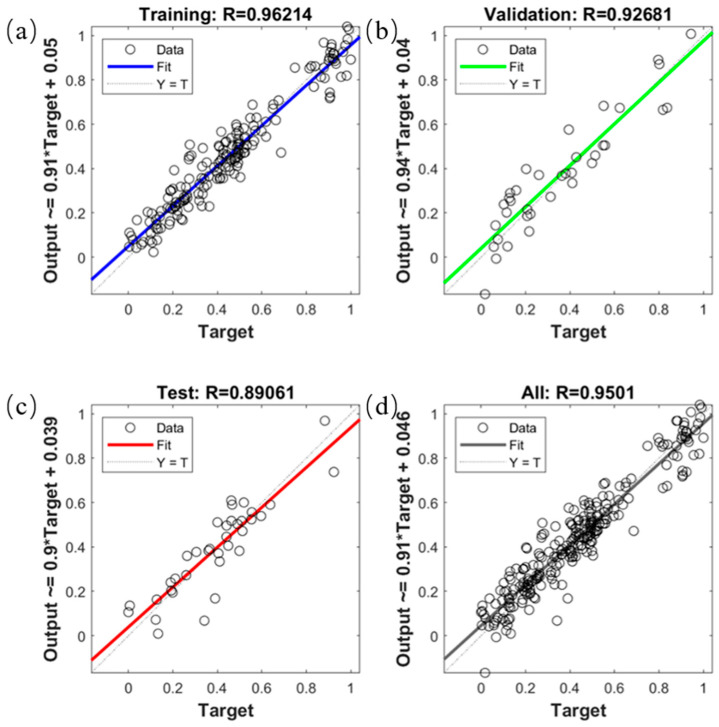
Model performance for four data sets in MSC-SPA-BP model regression. (**a**) Training set model performance; (**b**) validation set model performance; (**c**) test set model performance; (**d**) all data set model performance.

The model performance of the four data sets in the MSC-SPA-BP model regression is shown in Figure 6. Regarding the performance of the model in MSC-SPA-BP regression for the training set, validation set, test set, and all data sets, the regression values were 0.9621, 0.9268, 0.8906, and 0.9501, respectively, which indicates that the model performance was good.

**Figure 7:**
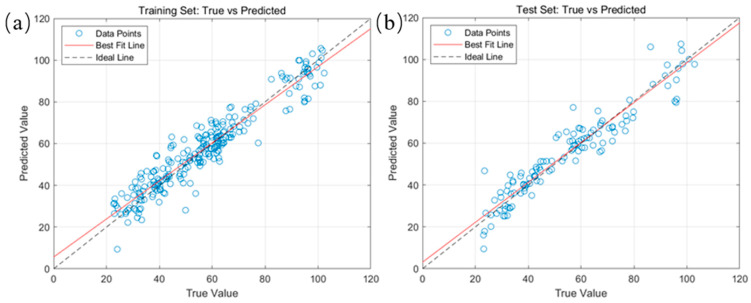
Prediction results of the optimal MSC-SPA-BP model for fatty acid values of corn. (**a**) Linear relationship between the predicted and true values of the training set; (**b**) linear relationship between the predicted and true values of the test set.

The prediction results of the optimal MSC-SPA-BP model for the fatty acid values of corn based on the SPA feature band are shown in Figure 7. It can be seen that the scatter points of the training set and the test set were basically distributed on both sides of their corresponding fitting curves and that the corresponding fitting curves were basically coincident with the X = Y line. This shows that the training and test values showed a good linear relationship with their corresponding predicted values, which means that the MSC-SPA-BP model had a good prediction ability for fatty acid values and can meet the requirements of the application.

In summary, the best model for the neural network prediction of fatty acid values in the full-band range was MSC-BP, with R^2^ = 0.8617 and RMSE_P_ = 8.8030 for the test set. The best model for predicting fatty acid values under the CARS-based eigen band was SG2-CARS-BP, with a coefficient of determination of the test set of R^2^ = 0.8548, which was a reduction of 0.0069 compared to the best model of the full band, MSC-BP, and the RMSE_P_ = 8.4467, which was a reduction of 0.3563 compared to the best model of the full band, MSC-BP. A total of 40 wavelengths were extracted from the SG2-CARS-BP model, accounting for 15.63% of the total number of wavelengths. However, based on the best model for predicting fatty acid values under the SPA feature band, which was MSC-SPA-BP, the coefficient of determination of the test set (R^2^) was 0.8949, which was an increase of 0.0332 compared to the best model MSC-BP for the full band, and the RMSE_P_ = 7.0581, which was a decrease of 1.7449 compared to the best model MSC-BP for the full band. A total of 24 wavelengths were extracted from the MSC-SPA-BP model, which was only 9.38% of the total number of wavelengths.

### 2.4. Model Construction for Predicting Fatty Acid Values of Corn Based on Random Forest

Full-band and eigen-band modeling were established based on random forest (RF) fatty acid values combined with RAW, SG, SG1, SG2, D1, D2, MSC, and SNV preprocessed spectra. The prediction information is shown in Table 3. It can be concluded that the highest coefficient of determination for the training set was the SG2-SPA-RF model, and the highest coefficient of determination for the test set was the SG2-SPA-RF model; thus, the best prediction model was the SG2-SPA-RF model.

**Figure 8:**
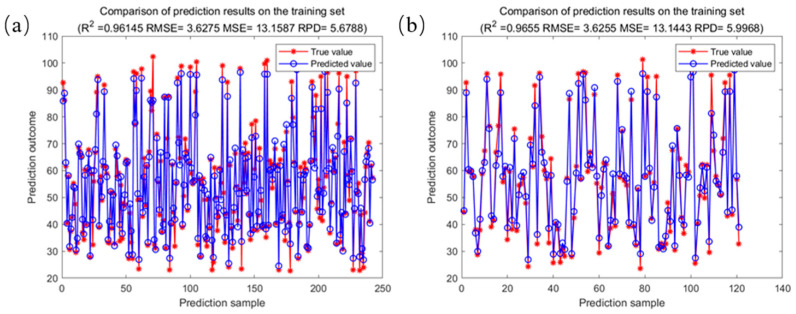
Results of the analysis of fatty acid values of corn based on the best SG2-SPA-RF model. (**a**) Comparison of prediction results for the SG2-SPA-RF training set; (**b**) comparison of prediction results for the SG2-SPA-RF test set.

The results of the analysis of fatty acid values of corn based on the SG2-SPA-RF model are shown in Figure 8. Figure 8a shows the comparison of the training set to fatty acid value modeling prediction results, where the training set coefficient of determination was 0.9615, the RMSE was 3.6275, and the fatty acid value of the corn samples was predicted with the best trained SG2-SPA-RF prediction model. Figure 8b shows the comparison of the prediction results of the prediction set for modeling the fatty acid values, with a test set coefficient of determination of 0.9655 and RMSE of 3.6255.

**Figure 9:**
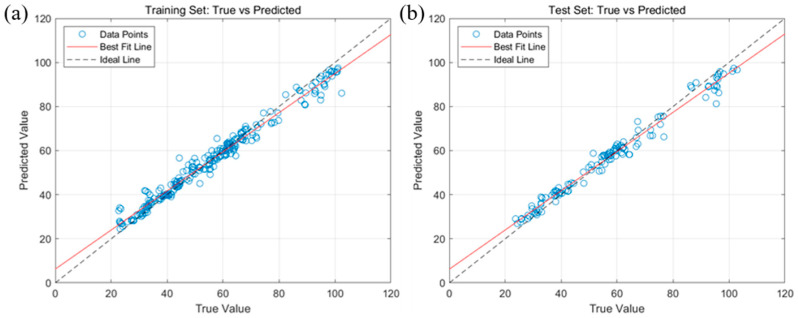
Prediction results of the optimal SG2-SPA-RF model for fatty acid values of corn. (**a**) Linear relationship between the predicted and true values of the training set; (**b**) linear relationship between the predicted and true values of the test set.

The prediction results of the optimal SG2-SPA-RF model for the characteristic bands of fatty acid values of maize are shown in Figure 9. Compared with Figure 7, this model fitted both the training and test sets better than MSC-SPA-BP, and its scatter distribution was more uniform. Therefore, the SG2-SPA-RF model had a better prediction ability for fatty acid values.

In summary, the best model for RF to predict fatty acid values in the full-band range was SG2-RF, with a coefficient of determination of 0.9567 and RMSE of 3.8408 for the test set. The best model for RF to predict fatty acid values under the CARS feature band was SG1-CARS-RF, with a coefficient of determination of 0.9504 for the test set, which was a decrease of 0.0063, and an RMSE of 4.1216, which increased by 0.2808 compared to the full-band best model, SG2-RF. A total of 38 wavelengths were extracted by the SG1-CARS-RF model, which accounted for 14.84% of the total wavelengths. However, under the SPA feature band, the best model for predicting fatty acid values by RF was SG2-SPA-RF, which had a coefficient of determination of 0.9655 for the test set, which was an increase of 0.0088 compared to the best model SG2-RF for the full band, and an RMSE of 3.6255, which was an increase of 0.2153 compared to the best model SG2-RF for the full band. SG2-SPA-RF 31 wavelengths were extracted, and were only 12.11% of the total number of wavelengths.

### 2.5. Visualization of Fatty Acid Values in Corn

**Figure 10:**
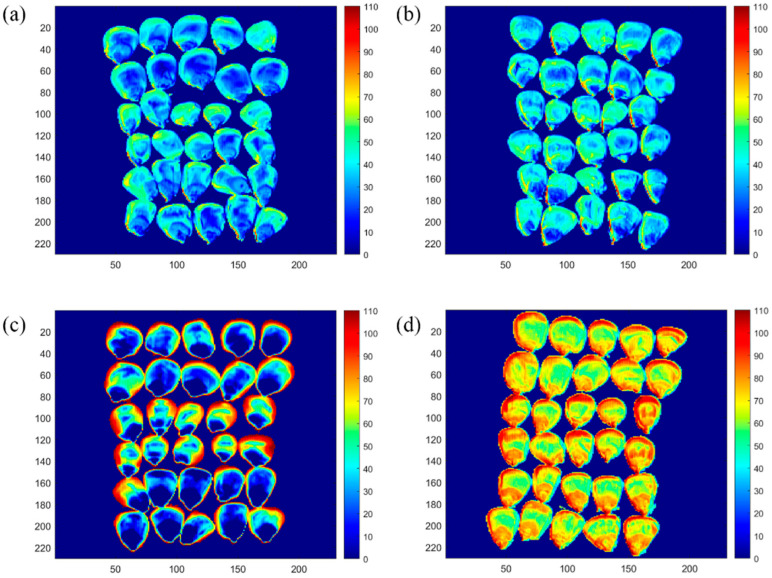
Visual distribution of fatty acid values of corn aged new. (**a**) Visual distribution of fatty acid values of corn aged 0 days; (**b**) visual distribution of fatty acid values of corn aged 70 days; (**c**) visual distribution of fatty acid values of corn aged 140 days; (**d**) visual distribution of fatty acid values of corn aged 210 days.

The pseudo-color visualization of the distribution of fatty acid values of 30 Zhengdan 958 corn kernels aged for 0, 70, 140, and 210 days is shown in Figure 10, respectively. The closer the color of this distribution graph is to red, the higher the value of fatty acids in the corn kernel, and the closer the color is to blue, the lower the value of fatty acids in the corn kernel. In Figure 10a, 30 images of Zhengdan 958 kernels have about 50% of blue areas and about 50% of the sum of yellow and green areas. At this time, the fatty acid value of the corn kernel was 33.25 mg KOH/100 g; thus, the corn kernel was judged to be new. From Figure 10b, it can be seen that 30 Zhengdan 958 kernel images comprise 30% of the blue areas, the image of the sum of green and yellow areas account for about 70%, and the image appears in a very small part of the red area. At this time, the fatty acid value of the corn kernel was 41.91 mg KOH/100 g; thus, the corn kernel was judged to be newer. In Figure 10c, it can be seen that 30 Zhengdan 958 kernel images comprise about 30% of the blue-biased areas, and the sum of the red, green, and yellow areas in the figure account for about 70%. At this time, the fatty acid value of the corn kernel was 56.87 mg KOH/100 g; thus, the corn kernel was judged to be more aged. In Figure 10d, 30 images of Zhengdan 958 kernels have 30% reddish areas and the sum of green and yellow areas in the figure is about 40%. At this time, the fatty acid value of the corn kernel was 71.91 mg KOH/100 g; thus, the corn kernel was judged to be aged. The fatty acid values estimated from the visualization plots were approximately the same as the fatty acid values measured in the experiment, indicating that the visualization plots can estimate the magnitude of the fatty acid values more accurately. Thus, it is possible to quickly and non-destructively discriminate whether a corn kernel is fresh and new-aged based on the visual distribution of its fatty acid values.

## 3. Materials and Methods

### 3.1. Test Materials

Three varieties of corn kernels: Yuyu 30 and Dedan 5 from Henan Qiule Seed Industry Co. and Zhengdan 958 from Luoyang, Henan, China.

### 3.2. Sample Processing

First, the kernels of three corn varieties, Dudan 5, Yuyu 30, and Zhengdan 958, were individually de-hybridized to select intact kernels. Secondly, the selected corn kernels were separately packed into breathable non-woven bags and each variety of kernel was divided into 160 portions of 200 g each. Finally, these corn samples were placed in an intelligent constant temperature and humidity incubator (LRH-350) set at a storage temperature of 40 °C and a relative humidity of 90% RH for accelerated aging [34]. Samples were taken at 7-day intervals, with 12 samples removed each time, for a total of 32 times, for a storage period of 217 days, with a total of 384 samples removed. After each sample was taken, hyperspectral images were first acquired, then fatty acid values were determined, and finally the remaining samples were placed in a −80 °C refrigerator for later use in corn sample visualization.

### 3.3. Determination of Fatty Acid Values

The fatty acid value in corn was determined by GB/T 5510-2011. The main principles of this standard for determining fatty acid values were as follows: extract the free fatty acids in the oil with petroleum ether by oscillation, and add ethanol solution after standing and filtering. Use phenolphthalein as indicator and titrate with potassium hydroxide standard titration solution. Determine the titration end point according to the color change of the lower solution. Calculate the fatty acid value from the volume of potassium hydroxide standard titration solution consumed. Take each sample four times and subject each sample to two parallel determinations of fatty acid values.

### 3.4. Image Acquisition and Correction for Hyperspectral Image

The corn samples were removed from the intelligent constant temperature and humidity chamber and left at room temperature to bring the sample temperature back to room temperature. The hyperspectral imaging system (Gaiasorter-Dual) was turned on to warm up for 0.5 h. The embryonic side of the corn kernel was brought down and the corresponding image was captured. The camera was made to focus on the corn samples for the focusing process, first through the camera elevator to adjust the height of the camera for coarse adjustment of the focus, and then through the rotation of the camera lens for fine-tuning focus adjustment, in order for the acquisition of the curves to become the sharpest. Spec View (1.0) software was opened on our computer to set the parameters. In order to make the collected sample images become clear and undistorted, the optimal corn seed collection parameters were finalized after repeated tests: the camera exposure time was set to 11.2 ms, the electronically controlled moving platform’s forward and backward speeds were set to 0.5 cm/s and 2 cm/s, respectively, the camera height was 13.5 cm, the starting position (Abs) was 54 cm, and the distances between the objective lens and the platform were both 40 cm [34].

In order to reduce the effects and noise due to physical differences in the detector sensitivity and camera as well as external interferences, the acquired raw hyperspectral image (*R*_0_) needs to be black-and-white-corrected to the reflectance spectral mode [23]. The blackboard image (*B*) was obtained by employing a black lens cap to cover the lens, which has a reflectance close to 0%. The whiteboard (*W*) was obtained by means of a PTFE sheet, whose reflectance is close to 100%. The corrected image (*R_C_*) was calculated by using the following equation:

$$R_{C}=\frac{R_{0}-B}{W-B}\times 100\%$$

### 3.5. Data Analysis

In ENVI 5.3 software, band math, build mask, and apply mask were utilized to build the mask and applied, and the ROI tool was used to select the region of interest (ROI) on the corrected hyperspectral image, where the ROI tool was used to select the ROI of the corrected hyperspectral image, and the average spectral data of the region of interest was calculated and extracted [35]. In MATLAB (2020b) software, all the spectral data of three different samples were subjected to the Mahalanobis distance to reject the anomalous data, and then the rejected data of the three different samples were integrated together to reject the anomalous values again. Finally, the rejected spectral data were processed and modeled.

In this study, seven preprocessing methods were used to preprocess the spectral data, including the convolutional smoothing derivative method (SG, SG1, SG2), derivative method (D1, D2), multivariate scattering correction (MSC), and standard normal transform (SNV) [36,37], in order to improve the data quality, reduce the noise interference, and extract the characteristic information. Competitive adaptive re-weighted sampling (CARS) and the sequential projection algorithm (SPA) were used to extract the characteristic wavelengths associated with the fatty acid values of corn [38,39,40]. Two methods, a BP neural network (BPNN) and random forest (RF), were used to classify and regressively analyze the spectral data and to build a model to predict the fatty acid values of corn [41,42,43].

### 3.6. Visualization of Fatty Acid Values of Corn

**Figure 11:**
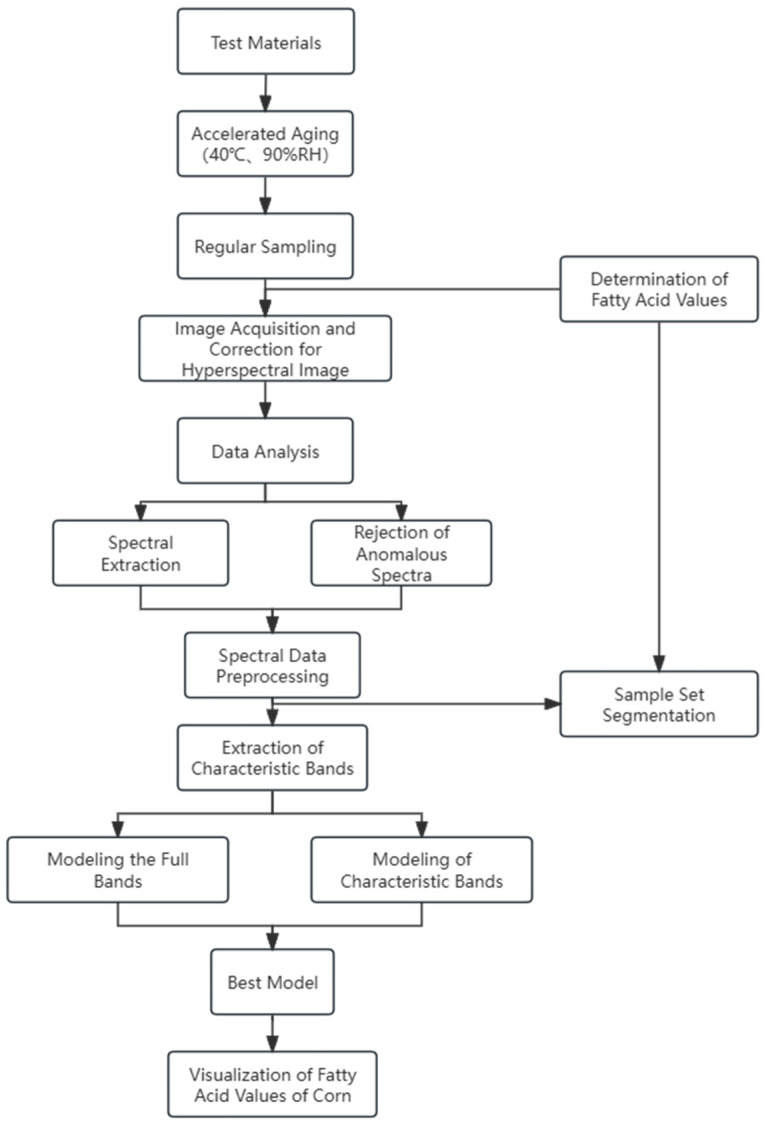
Technology roadmap.

Corn hyperspectral images of three varieties with aging times of day 0, 70, 140, and 210 were re-collected individually to visualize chemical information on corn fatty acid values. In order to represent all the information of the corn kernel spectral image, a square area of 230 × 230 = 52,900 pixel points was selected for spectral data extraction and prediction. The developed optimal model SG2-SPA-RF was used to predict the fatty acid values of corn and was plotted as pseudo-colored visual distribution images [44]. The technology roadmap is shown in Figure 11.

## 4. Conclusions

Hyperspectral imaging technology, combined with the BPNN and RF machine learning method, successfully predicted the fatty acid values of corn and characterized the aging of corn. The experimental results showed that the fatty acid values can be an important index for judging the new aging degree of corn. The SG2-SPA-RF model was the best for the quantitative prediction of fatty acids in corn. The coefficient of determination was 0.9655 and the root mean square error was 3.6255. Moreover, the best obtained SG2-SPA-RF model was used to transfer the spectrum of each pixel into its corn fatty acid values; thus, visualization maps of the fatty acid values distribution were generated. The results of this study indicate that the HSI has a good predictive ability and potential for corn fatty acid values. At the same time, the visualization of the distribution map is useful for quickly determining the freshness of the stored corn. Experimental results show that this method is feasible. This method can be applied to the quality detection of more varieties of corn and other grains in the follow-up study, which provides theoretical support for rapid non-destructive testing.

## Figures and Tables

**Table 1:** Results of the sample division of fatty acid values of corn.

Sample Set	Sample Size	Fatty Acid Value (mg KOH/100 g)			
		Maximum	Minimum	Average Value	Standard Deviation
Training set	242	102.93	22.68	58.44	21.87
Test set	121	98.49	23.48	51.85	18.55

**Table 2:** Results of statistical analysis for predicting fatty acid values of corn based on BPNN full waveband.

Pretreatment	Training Set			Test Set		
	Rc^2^	RMSEc	MAPEc	Rp^2^	RMSEc	MAPEc
RAW-BP	0.8660	5.9845	0.3170	0.6798	0.7438	0.4553
SG-BP	0.8858	6.2572	0.0982	0.8293	7.7727	0.1266
SG1-BP	0.8613	7.7252	0.1312	0.8445	8.3271	0.1377
SG2-BP	0.9107	6.0349	0.0899	0.7208	10.9412	0.1361
D1-BP	0.9315	5.5645	0.0536	0.8150	8.7462	0.1263
D2-BP	0.9093	6.2641	0.0763	0.6989	13.0575	0.1853
MSC-BP	0.8387	8.0021	0.1165	0.8617	8.8030	0.1455
SNV-BP	0.9297	5.4476	0.0757	0.8218	8.6234	0.1240
RAW-CARS-BP	0.8119	6.7643	0.4312	0.6915	9.5862	0.5957
SG-CARS-BP	0.8685	7.9494	0.1166	0.8394	9.1946	0.1381
SG1-CARS-BP	0.9043	6.2501	0.0917	0.8122	8.5736	0.1384
SG2-CARS-BP	0.9204	5.5003	0.0770	0.8548	8.4467	0.1210
D1-CARS-BP	0.7980	8.2932	0.4819	0.7972	8.5857	0.5528
D2-CARS-BP	0.8645	5.8363	0.2916	0.7015	9.7550	0.4691
MSC-CARS-BP	0.8236	6.5603	0.2478	0.8017	7.0142	0.3599
SNV-CARS-BP	0.8226	6.0610	0.3097	0.8214	6.9934	0.3574
RAW-SPA-BP	0.8506	8.1238	0.1287	0.7090	11.1226	0.1624
SG-SPA-BP	0.8819	6.4897	0.1037	0.8262	8.4327	0.1258
SG1-SPA-BP	0.9241	5.5883	0.0838	0.8820	7.5515	0.1191
SG2-SPA-BP	0.8945	6.4355	0.0989	0.7627	8.5817	0.1377
D1-SPA-BP	0.9089	6.1796	0.07762	0.8705	7.5989	0.1033
D2-SPA-BP	0.8437	7.9903	0.0959	0.6319	14.2419	0.2258
MSC-SPA-BP	0.8933	6.4775	0.1014	0.8949	7.0581	0.1076
SNV-SPA-BP	0.7989	8.5473	0.1311	0.7074	9.017	0.1428

**Table 3:** Results of statistical analysis for predicting fatty acid values of corn based on RF full waveband.

Pretreatment	Training Set			Test Set		
	Rc^2^	RMSEc	MAPEc	Rp^2^	RMSEc	MAPEc
RAW-RF	0.8828	8.9837	0.0911	0.8460	6.5106	0.0979
SG-RF	0.8689	6.1217	0.0920	0.8534	6.6442	0.0990
SG1-RF	0.9695	3.4059	0.0481	0.9354	4.1953	0.0626
SG2-RF	0.9537	3.9452	0.0589	0.9567	3.8408	0.0567
D1-RF	0.9669	3.4372	0.0476	0.9467	4.0964	0.0578
D2-RF	0.9247	4.4579	0.0682	0.9017	5.7885	0.0922
MSC-RF	0.9338	4.6645	0.0649	0.8727	6.0067	0.0953
SNV-RF	0.9304	4.7527	0.0689	0.8984	5.6229	0.0854
RAW-CARS-RF	0.8828	5.8947	0.0872	0.8440	6.9076	0.1089
SG-CARS-RF	0.8703	6.1707	0.0910	0.8641	6.4221	0.0996
SG1-CARS-RF	0.9562	3.8170	0.0554	0.9504	4.1216	0.0603
SG2-CARS-RF	0.9538	3.9964	0.0569	0.9196	4.9458	0.0746
D1-CARS-RF	0.9555	3.9768	0.0497	0.9250	4.7160	0.7012
D2-CARS-RF	0.9103	4.8881	0.0738	0.9018	5.8638	0.0972
MSC-CARS-RF	0.9237	4.9561	0.0715	0.9068	5.2608	0.0779
SNV-CARS-RF	0.9224	5.0055	0.0710	0.9176	5.2332	0.0759
RAW-SPA-RF	0.8425	6.6777	0.1035	0.8523	6.4739	0.1030
SG-SPA-RF	0.8564	6.2990	0.0973	0.8052	7.4769	0.1181
SG1-SPA-RF	0.9715	3.1123	0.0408	0.9134	4.9158	0.0695
SG2-SPA-RF	0.9615	3.6275	0.0520	0.9655	3.6255	0.0523
D1-SPA-RF	0.9617	3.5357	0.04816	0.9462	4.5827	0.0659
D2-SPA-RF	0.9015	5.5503	0.0884	0.8338	5.4602	0.0898
MSC-SPA-RF	0.9168	5.2789	0.0733	0.9202	4.8252	0.0716
SNV-SPA-RF	0.9224	5.0055	0.0710	0.9176	5.2332	0.0759

## Data Availability

The data presented in this study are available on request from the corresponding author.
